# Comparison of Equations Estimating Resting Metabolic Rate in Older Adults with Type 2 Diabetes

**DOI:** 10.3390/jcm10081644

**Published:** 2021-04-12

**Authors:** Assaf Buch, Jonathan Diener, Naftali Stern, Amir Rubin, Ofer Kis, Yael Sofer, Mariana Yaron, Yona Greenman, Roy Eldor, Sigal Eilat-Adar

**Affiliations:** 1 Institute of Endocrinology, Metabolism and Hypertension, Tel Aviv Sourasky Medical Center, Tel-Aviv 64239, Israel; oferkis58@gmail.com (O.K.); yaelso@tlvmc.gov.il (Y.S.); marianay@tlvmc.gov.il (M.Y.); yonagr@tlvmc.gov.il (Y.G.); roye@tlvmc.gov.il (R.E.); 2 The Sagol Center for Epigenetics of Metabolism and Aging, Tel Aviv Sourasky Medical Center, Tel-Aviv 64239, Israel; naftalis@tlvmc.gov.il; 3 School of Health Sciences, Ashkelon Academic College, Ashkelon 78211, Israel; 4 Institute of Sports and Sports Science, Karlsruhe Institute of Technology, 76131 Karlsruhe, Germany; jonathan.diener@web.de; 5 The Academic College at Wingate, Wingate Institute, Netanya 42902, Israel; amirsportdiet@gmail.com (A.R.); sigaleilat70@gmail.com (S.E.-A.); 6 The Sackler Faculty of Medicine, Tel-Aviv University, Tel-Aviv 69978, Israel

**Keywords:** resting metabolic rate, elderly, type 2 diabetes, obesity, predictive equations, energy expenditure

## Abstract

Measuring resting metabolic rate (RMR) is time-consuming and expensive, and thus various equations for estimating RMR have been developed. This study’s objective was to compare five equations in elderly people with type 2 diabetes (T2DM). RMR was measured in 90 older adults (≥65 years) with T2DM (mean body mass index (BMI) of 31.5 kg/m^2^), using indirect calorimetry. Results were compared to four frequently used equations (those of Cunningham, Harris and Benedict, and Gougeon developed for young adults with T2DM, and that of Lührmann, which was developed for the elderly), in addition to a new equation developed recently at the Academic College at Wingate (Nachmani) for overweight individuals. Estimation accuracy was defined as the percentage of subjects with calculated RMR within ±10% of measured RMR. Measured RMR was significantly underestimated by all equations. The equations of Nachmani and Lührmann had the best estimation accuracy: 71.4% in males and 50.9% in females. Skeletal muscle mass, fat mass, hemoglobin A1c (HbA1c), and the use of insulin explained 70.6% of the variability in measured RMR. RMR in elderly participants with T2DM was higher than that calculated using existing equations. The most accurate equations for this specific population were those developed for obesity or the elderly. Unbalanced T2DM may increase caloric demands in the elderly. It is recommended to adjust the RMR equations used for the target population.

## 1. Introduction

The continuous spread of type 2 diabetes mellitus (T2DM) represents a global health threat. It is estimated that 463 million people worldwide had diabetes in 2019, with a predicted increase to 700 million by 2045 [1]. The main reasons for the rising numbers of T2DM cases lie in the increased prevalence of obesity, as well as unhealthy eating habits, sedentary behavior, and an aging population [2]. The results of Schellenberg et al. [3] suggest that many cases of T2DM could be prevented by 30 min of daily physical activity (PA), the maintenance of a healthy body weight, and healthy diet. The daily total energy expenditure (TEE) is defined as the sum of the resting metabolic rate (RMR), the thermic effect of food, and physical activity energy expenditure. RMR, defined as the minimal rate of metabolism necessary to maintain life, accounts for 60–70% of TEE in most individuals [4]. In order to lose weight, a lower caloric intake compared with energy expenditure is required; therefore, an assessment of energy expenditure is needed. Commonly, there are two methods applied to assess RMR. Measurement via indirect calorimetry (IC) is considered to be the reference standard (“gold standard”) [5]. Nevertheless, due to the need for special instruments and trained clinical staff (as well as the high cost), its application is restricted. Therefore, various equations have been developed over the years to estimate RMR for clinical practice [6]. Since various factors can influence RMR, including age, gender, body composition, ethnicity, physical activity, genetics, food intake, and the existence of obesity or T2DM [7], it is possible that adapted RMR equations for specific populations are needed.

Presumably, the most frequently used equation is that of Harris and Benedict (HB) [8], developed over 100 years ago using the RMR results of healthy Caucasian men and women with a broad age range. Another general equation, which focuses on the relationship of RMR and fat-free mass (FFM), was proposed by Cunningham (CU) [9]. He derived his equation from eight studies published in the 1980s, which included both normal-weight and obese subjects.

Body composition represented by weight as well as fat mass (FM) and fat-free mass (FFM) are factors influencing RMR; therefore, it seems advisable to use an equation derived from subjects similar to the population of interest in this respect. The equation of Nachmani et al. (NA [10]), developed at the Academic College at Wingate, was derived from subjects with a mean body mass index (BMI) of 32.5 kg/m^2^, and thus seems to be suitable for obese patients.

As mentioned above, age is another factor contributing to RMR. From the age of 20 years on, aging is linked to a progressive loss of 1–2% of RMR per decade [11]. Closely related to this decrease is the reduction in FFM [12]. It is still unclear whether the RMR decrease is exclusively due to changes in FFM. For instance, Krems et al. [13] observed a significantly lower RMR in the elderly compared to young subjects, despite adjustments for body composition. Hence, it might be advisable to use prediction equations derived from the data of subjects of similar age. Lührmann et al. (LU; [14]) developed a similar age-specific equation for older people using data from a large sample (*n* = 287). Caron et al. [15] suggested using the T2DM-adapted equation by Gougeon et al. (GO [16]) for T2DM patients, which includes fasting plasma glucose (FPG) as one of the explanatory variables.

To the best of our knowledge, there are no studies investigating the best-fit RMR equation for elderly people with T2DM. The main objective of the current study was to validate different equations by comparing measured with predicted RMR in T2DM patients aged 65 years or older. We hypothesized that the three specific equations (the GO, LU, and NA) would outperform the two general equations (HB and CU).

## 2. Materials and Methods

The present study is a cross-sectional analysis using baseline data from the Circuit training-Empagliflozin-Vegiterranean diet for older adults with type 2 diabetes (CEV65 study) [17], which compares the effectiveness of (1) circuit resistance training; (2) an antihyperglycemic drug (empagliflozin); and (3) a plant-based Mediterranean diet (“vegiterranean” diet) for delaying the progression from diabetes to sarcopenia and/or frailty. The recruitment process as well as the complete list of inclusion and exclusion criteria are described in detail elsewhere [17].

The current analysis includes data on 90 (out of 100) subjects who participated in the CEV65 trial with valid RMR measurements, as described in the next section. This is a post-hoc analysis from the original randomized CEV-65 trial where sample size was determined a priori as *n* = 120, with 100 participants recruited overall. The participants were older adults (aged ≥ 65 years) with low PA levels (≤ 2 days a week of any leisure aerobic PA), who were capable of walking independently with or without an assistive device (cane or walking aid). Participants were diagnosed with T2DM in accordance with American Diabetes Association guidelines [18] and treated with an antihyperglycemic agent other than a sodium glucose transporter (SGLT-2) inhibitor. Participants who were undergoing nutritional therapy, had recently (<1 month) changed their diet, had participated in a weight loss program, had performed resistance training, or had uncorrected hypothyroidism (thyroid-stimulating hormone (TSH) > 6 mL U/L) were excluded. FPG levels were obtained within 1 week prior to testing from clinics in health maintenance organizations. All the participants who volunteered for the CEV-65 trial were Jewish Caucasian residents of Tel Aviv and its area.

Written informed consent was obtained from all participants. The study protocol was approved by the TASMC Institutional Review Board and was registered a priori in ClinicalTrials.gov (NCT03560375). We ensured data confidentiality along the study by giving each participant a digit code representing him/her along the study. The codes were matched to the full names and details of the participants in a file which was stored in the server of the hospital, with access provided only to the primary researcher (A.B) and the study physician (R.E).

### 2.1. Measurement of RMR

RMR was measured by indirect calorimetry using an open-circuit ventilated canopy measurement device (Quark RMR; COSMED Srl, Rome, Italy). Its accuracy and reliability have been validated against a gold standard device (Deltatrec II; Sensormedics Corporation, Yorba Linda, CA, USA), which is no longer available [19,20]. The Quark RMR calculates RMR through the measurement of oxygen consumption (VO_2_) and carbon dioxide production (VCO_2_), together with other ventilatory parameters. The instrument was warmed up and calibrated automatically prior to each test session by using a gas mixture of 16% oxygen and 5% carbon dioxide, balanced with nitrogen.

Tests were performed with the participants in a supine position after a 5-min rest period in a dimly lighted room with a temperature of 21–22 °C. All measurements were conducted in the morning (08.30–10.00 a.m.) after a 12-h fast and 12-h restriction of any strenuous exercise. The participants were instructed to avoid speaking and to relax without falling asleep. Data were collected over a 10–15 min interval, where the first 5 min were used for familiarization and not counted. According to current recommendations, the RMR was considered valid if a steady state was achieved, defined as a 5-min period with less than 10% CV (coefficient of variation [SD × (mean of individual replicate measures) × 100]) for VO_2_ and VCO_2_ [21,22]. After reaching a steady state, measurements were stopped. In case a steady state was not reached, the test was aborted after 15 min to keep the subject burden as low as possible. The Weir equation [23] was used by the software to calculate RMR.

### 2.2. Anthropometric Measurements

Body mass was measured using an electronic scale with an accuracy of 0.1 kg. Body height was measured to the nearest 0.1 cm, with the subjects in light clothes and without shoes. FFM, skeletal muscle mass (SMM), and fat mass (FM) were evaluated by direct segmental multi-frequency bioelectrical impendence analysis (BIA) using the InBody 770 apparatus (InBody, Cerritos, CA, USA). The measurement suggested by the InBody apparatus was found to be a valid tool for the assessment of total body and segmental body composition [24].

### 2.3. Equations

The 5 equations with which the measured RMR (RMRm) was compared are presented in Table 1. As mentioned above, HB and CU equations were selected because they are commonly used in clinical practice. The other equations were chosen since they were derived from subjects of similar age (LU) who were also suffering from T2DM (GO) or had a similar body composition (NA).

### 2.4. Statistical Analysis

Results were analyzed with SPSS Statistics 25 for Windows (IBM, Armonk, NY, USA) and are expressed as the mean ± standard deviation (SD). Differences in participant characteristics between the males and the females were examined by unpaired *t*-test for continuous variables and the chi-squared test for categorical variables. A paired *t*-test was used to evaluate the mean difference between RMRm and the RMR estimated (RMRe) by equations. The mean difference was expressed as the absolute value (kcal/day, mean bias) and percentage (%, relative bias). Relative bias (%) was calculated as follows: Relative bias = (∆RMR_mean bias_)/RMRm × 100). Estimation accuracy was calculated for all 5 equations and was defined as the percentage of subjects whose RMRe was within ±10% of RMRm. Phang et al. [25] showed that this error limit of estimation accuracy was consistent with IC measurement errors of 5% or lower. Any other estimates were considered as errors and expressed as the percentage of participants whose RMR was underestimated (<90% RMRm) or overestimated (>110% RMRm) [26]. The chi-squared test was used to compare between them. The Bland–Altman test was used to assess any agreement between the calculated RMR and the ∆RMR_mean bias_. Linear regression was performed to find the variables that explained RMRm using stepwise methods. Variables entered into the regression were chosen based on their clinical relation to the metabolic state among diabetic and older adults and included: anthropometrics (SMM/FFM; FM), glycemic control (Hemoglobin A1c (HbA1c); insulin use (yes vs. no)), functional status (subjective functional status as a score, sit to stand 30-s test results, and polypharmacy defined as the use of >8 prescribed drugs (yes vs. no)). We made sure that there was no co-linearity between the confounders/predictors (over-adjustment) using a Spearman correlation matrix. We considered any between-confounder correlation >0.7 as indicative of co-linearity [27]. Results were regarded as statistically significant if the *p*-values were lower than 0.05.

## 3. Results

### 3.1. Characteristics of the Participants

Valid measurement was achieved in 90 participants, including 55 women and 35 men. Baseline characteristics by sex are described in Table 2. Participants were 70.4 ± 4.6 years old, had a mean weight of 83.9 ± 15.9 kg, and had a mean BMI of 31.5 ± 5.6 kg/m^2^. Age, BMI, FM, smoking, and FGP did not differ between men and women, while the percentage of FM was lower in the men and FFM was lower in the women.

### 3.2. Measured RMR as Compared to Predicted RMR

**Figure 1:**
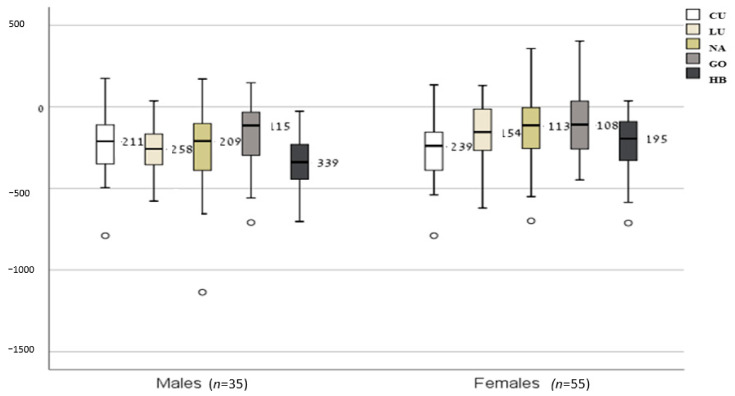
Mean bias between RMRe and RMRm (kcal/day) (presented as the median and interquartile difference). Box plots showing the median differences (inner line within the box), interquartile range (IQR) (Q1–Q3), and lines indicating variability outside the upper and lower quartiles (minimum = Q1 − 1.5*IQR; maximum = Q3 + 1.5*IQR) when RMR equations were compared to measured RMR. Abbreviations: RMRm = resting metabolic rate measured; RMRe = RMR estimated by equation; HB = Harris and Benedict [8]; LU = Lührmann et al. [14]; NA = Nachmani et al. [10]; CU = Cunningham [9]; GO = Gougeon et al. [16].

Mean RMRm was 1585 ± 214 kcal/day in females and 1888 ± 259 kcal/day in males. Mean RMRm differed significantly from all mean RMRe. In total, all equations, except for the NA equation in males, underestimated RMR significantly compared to RMRm (Table 3). ∆RMR_mean bias_ (median bias) values are shown in Figure 1. The lowest median bias (RMRe-RMRm) was observed for GO (male: −115 kcal/day, female: −108 kcal/day), followed by NA (male: −209 kcal/day, female: −113 kcal/day). The highest median difference was found for the HB and CU formulas among males and females, respectively.

Estimation accuracy is presented in Table 4. The equations of NA and LU performed best, with 59% accurate estimates, ahead of that of GO, with 52% accurate estimates. HB and CU achieved 43.3% and 25.6% accuracy, respectively.

**Figure 2:**
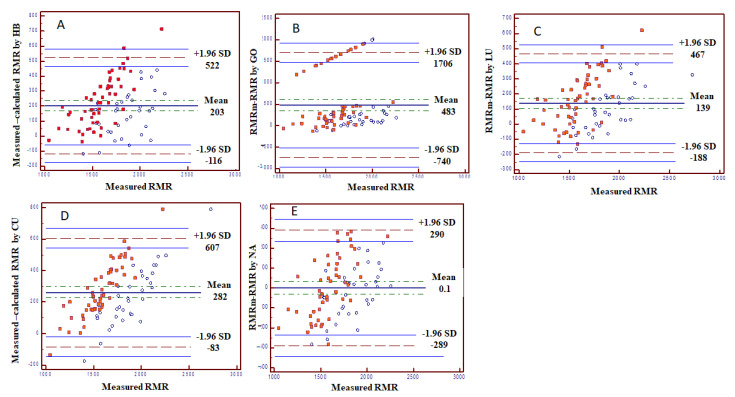
Bland-Altman plots for each equation. Bland–Altman plots showing the agreement between measured and calculated RMR (different formulas). (**A**) HB equation. (**B**) GO equation. (**C**) LU equation. (**D**) CU equation. (**E**) NA equation. The difference between the measured and calculated RMR (Y axis) is shown as a function of measured RMR. Fully colored circles represent females; unfilled circles represent males. Abbreviations: RMRm = resting metabolic rate measured; RMRe = RMR estimated by equation; HB = Harris and Benedict [8]; LU = Lührmann et al. [14]; NA = Nachmani et al. [10]; CU = Cunningham [9]; GO = Gougeon et al. [16].

Bland-Altman plots are presented in Figure 2, showing correlations between RMRm and ∆RMR_mean bias_. In all equations, the ∆RMR_mean bias_ increased as the RMRm was higher, indicating that there was no stable agreement between the RMRe and RMRm. However, the equations of NA and LU had the lowest ∆RMR_mean bias._

### 3.3. Variables Predicting Measured RMR in Older Adults with Diabetes

There was a clear co-linearity between SMM and FFM (r = 0.998), and both were highly correlated with RMRm (r = 0.76 for both) (data not shown). Therefore, only SMM was entered to the model. Using stepwise linear regression, the variables that explained the RMRm were SMM, FM, and HbA1C and whether receiving insulin (yes or no).

The final formula was:

(1) RMRm= 34.207∗SMM+ 7.091∗FM+38.199∗HbA1C+94.031∗Insulin use 

The variables which entered the model explained 57.8%, 7.2%, 3.7%, and 2% of the variability, respectively (total: 70.6%).

## 4. Discussion

In the present study, the RMR of older patients with T2DM measured by indirect calorimetry was compared to the RMR estimated by five different equations. Estimating RMR in this population is important due to the increasing prevalence of overweight and obesity and the consequent rising number of individuals suffering from T2DM, as well as the aging of the population worldwide. However, all the examined equations underestimated RMR, and their inaccuracy was in a larger proportion in women as compared to men. Only in male participants were the NA and LU equations able to achieve accurate results (in approximately 70% of the individuals).

Larger individual errors might be covered by group mean data because high positive errors may counterbalance high negative errors. Therefore, they cannot be utilized for practical decisions in individual patients [26]. For this reason, there is a need to define variables that may affect RMR in this specific population.

Gougeon et al. [16] showed that weight was the main determinant of RMR for patients with obesity and T2DM. Hence, it seems likely that the NA equation along with the LU equation (which are based on a similar sample) will have a better fit. Nevertheless, even the NA equation had a relatively weak estimation accuracy, especially in women. Since the NA equation has only recently been developed and is not yet validated in other populations, further studies are needed to evaluate its applicability to older T2DM patients.

Although the sample from which the LU equation was derived had substantial differences in body composition compared to their counterparts in the current study, it was based on people of a similar age. This equation had the same estimation accuracy as that of NA.

Noreik et al. [28] also found the LU equation to be the most accurate for estimating RMR (mean ± SD ∆RMR_mean bias_: 25.8 ± 99.4 kcal/day) in their study with obese older subjects. This supports the findings of Krems et al. [13], indicating that age might be a factor which should be considered in RMR estimation.

Gougeon et al. [16] showed that implementing FPG improved the validity of their proposed equation by 3%. Surprisingly, the GO equation integrating glycemic status did not provide accurate estimates for either sex in our study. This result contradicts the finding of the review by Caron et al. [15] that GO is the best choice for estimating RMR in diabetes patients, although this conclusion is based on only two studies. An explanation for the underestimation of RMR in the current study might be the better glucose control in the current study. The mean FPG value of 7.8 mmol/L is only slightly above the American Diabetes Association’s treatment goals for glycemia in older adults with diabetes and/or a few coexisting chronic illnesses, indicating a stable T2DM in most subjects [29]. Ryan et al. [30] compared the RMR in obese patients without diabetes to that of obese, mildly glycemic diabetics. RMR (absolute and adjusted) was unaffected by glucose levels, with FFM being the only determinant of RMR. However, when comparing diabetics with FPG values > 10 mmol/L to diabetics with FPG values > 10 mmol/L, Gougeon et al. [16] found a significantly higher RMR in the group with higher FPG values when corrected for weight, FFM, age, and gender. In the current study the impact of glycemic control on RMR was expressed by higher HbA1c levels predicting higher RMR levels, and by the use of insulin. A possible metabolic explanation may be an increased glycosuria and/or gluconeogenesis caused by hyperglycemia [31,32], in which glucose is produced from non-carbohydrate substrates [32]. A concentration of 10 mmol/L is considered the threshold above which patients experience glycosuria [33], which is a mechanism for eliminating excess circulating glucose through the urine [31]. Therefore, the GO equation may not be suitable for T2DM patients with treated and stable DM.

The general HB and CU equations performed considerably worse compared to the specific equations. Both underestimated RMR significantly by a mean of more than 200 kcal/day and could only obtain accurate measurements in well below 50% of the subjects. The results of other studies were inconclusive. In some studies, the HB equation significantly overestimated RMR [16,34,35,36]. Frankenfield et al. [26] also found an overestimation of RMR in obese subjects—up to 62%. In two studies no differences between measured and estimated RMR by HB equations were found. [37,38]. Only Huang et al. [39] reported an underestimation of 3.1% in women by the HB equation. Frankenfield et al. [27] also concluded in their review of RMR estimation in obese subjects that overestimation errors of the HB equation were reported more frequently than underestimation errors in all studies included. Accurate results were achieved in 38% to 64% of obese subjects. However, when examining healthy older subjects (mean age over 60 years), Fredrix et al. [40], Lührmann et al. [14], and Melzer et al. [41] reported a significant underestimation of RMRm in both sexes, as did Arciero et al. [42] and Itoi et al. [43] in male subjects.

Supported by the Bland-Altman test results, it is possible that inaccuracies between the predicted and measured RMR were derived mainly from participants with higher RMR levels. This was expressed by the fact that there was no consistency in the correlation between the ∆RMR_mean bias_ and RMRm, whereas a higher RMRm was correlated with a larger RMR difference. A population-specific equation is suggested based on the current sample, which includes traditional variables explaining differences in RMR such as SMM and FM (which represent weight, and specific variables for diabetes such as the use of insulin and HbA1c.)

The current study has several limitations. The recruited subjects do not represent all older adults with T2DM, resulting in a possible selection bias that could have influenced external validity. However, for relatively balanced and young/older adults with T2DM, the current suggested equation may be valid. A larger sample size could have resulted in a better understanding of the reasons for the inaccuracies of the equations using data stratifications.

## 5. Conclusions

To the best of our knowledge this is the first study examining the accuracy of common equations predicting RMR in older adults with T2DM. The population-specific equations performed substantially better than the two general equations. On the group level, the NA equation produced valid estimates of RMR in males. However, on the individual level none of the equations could achieve accurate estimates of RMR in more than 60% of the subjects. Especially in women, the estimation accuracy was found to be weak. If measurement is not possible, equations derived from a sample as similar as possible (including glycemic status) should be used for older subjects with T2DM. Further research is needed to clarify how the presence of T2DM affects RMR.

## Figures and Tables

**Table 1:** RMR equations.

Author(s)	Year	Cohort Characteristics	Equation
Cunningham [9]	1991	*n* = 1483; meta-analysis, normal-weight and obese males and females, did not provide detailed age information	RMR = 370 + 21.6 FFM
Harris and Benedict [8]	1919	*n* = 239 (136 M, 103 F) Age = 29 ± 14 years	Male: RMR = 66.47 + 13.75 Wt + 5.00 Ht − 6.76 A Female: RMR = 655.09 + 9.56 Wt + 1.85 Ht − 4.68 A
Gougeon et al. [16]	2002	*n* = 65; with type 2 DM 40 F (age = 52 ± 1, BMI = 37 ± 1, FPG = 10.9 ± 0.5) 25 M (age = 54 ± 2, BMI = 37 ± 1, FPG = 10.0 ± 0.9)	RMR = 375 + 85 Wt − 48 FM + 63 FPG
Lührmann et al. [14]	2002	*n* = 281; over 60 years of age 179 F (age = 67.8 ± 5.7, BMI = 26.4 ± 3.7) 107 M (age = 66.9 ± 5.1, BMI = 26.3 ± 3.1)	RMR = 757 + 11.9 Wt − 3.7 A + 178 S
Nachmani et al. [10]	2020	*n* = 39 18 F (age = 45.5 ± 12.5, BMI = 32.0 ± 4.0) 21 M (age = 38.2 ± 11.0, BMI = 33.0 ± 3.0)	Male: RMR = 132.82 + 28.37 Wt − 205.59 Ht + 9.46 FFM − 2.87 A − 25.93 FM Female: RMR = 553.97 + 16.60 Wt + 1033.84 Ht − 13.73 FFM − 10.93 A − 19.67 FM

**Note:** Abbreviations: A = age (years); BMI = body mass index (kg/m^2^); Ht = height (cm; Nachmani equation = m); F = female; FPG = fasting plasma glucose (mmol/L); FFM = fat-free mass (kg); FM = fat mass (kg); M = male; Wt = weight (kg); RMR = resting metabolic rate (kcal/day); S = sex (female = 0, male = 1).

**Table 2:** Baseline characteristics (means ± SD for continuous variables, *n* (%) for categorical variables).

Variable	Males (*n* = 35)	Females (*n* = 55)	*p* Value (Males vs. Females)
Age (years)	70.3 ± 4.2	70.4 ±4.8	0.951
Ht (cm)	172.0 ± 6.4 *	158.5 ± 4.5 *	<0.001
Wt (kg)	93.3 ± 15.1 *	77.9 ± 13.3 *	<0.001
BMI (kg/m^2^)	31.6 ± 5.1	31.1 ± 5.6	0.658
FM (kg)	34.0 ± 11.2	34.5 ± 9.7	0.809
FM (%)	35.7 ± 6.4 *	43.5 ± 5.8 *	<0.001
FFM (kg)	59.3 ± 6.2 *	43.4 ± 4.7 *	<0.001
Smokers	2 (5.7%)	2 (3.6%)	0.715
FPG (mmol/l)	8.5 ± 2.9 (*n* = 31)	7.6 ± 2.0 (*n* = 40)	0.157
HbA1c (%)	7.5 ± 1.4 (*n* = 34)	7.4 ± 1 (*n* = 51)	0.491
Insulin users (long and/or short)	7 (20%)	16 (29.1%)	0.335
Functional score (0–36) ^1^	11.9 ± 4.5	12.7 ± 3.9	0.382
Sit to stand 30-s test (number of repetitions)	11.2 ± 1.8	10.5 ± 3.1	0.156
Polypharmacy (>8 prescribed medications)	9 (25.7%)	15 (27.3%)	0.708

Note: ^1^ The Comprehensive Functional Assessment Questionnaire (CFAQ) was used to assess physical function state by questions on Activities of daily living (ADL) (9 questions in total). Each had an answer score of 0–4. A score of “0” indicates that the activity was not performed for reasons unrelated to health problems. An increasing score from 1 to 4 reflects greater difficulty associated with the specific activity. Answers for each of the 9 questions in the functional questionnaire were summed to a total score ranging from 0 (no impairment at all) to 36 (the highest functional impairment). * Significant difference (*p* < 0.05). Abbreviations: HbA1c = hemoglobin A1c; Ht = height (cm); Wt = weight (kg); BMI = body mass index (kg/m^2^); FFM = fat-free mass (kg); FM = fat mass (kg); FPG = fasting plasma glucose (mmol/l).

**Table 3:** Measured RMR (RMRm) compared to predicted RMR (RMRe) (means ± SD).

RMR	Males (*n* = 35) (kcal/day)	Females (*n* = 55) (kcal/day)	Total (*n* = 90) (kcal/day)
RMRm	1888 ± 259	1585 ± 214	1703 ± 275
RMRe (HB)	1734 ± 225 *	1350 ± 137 *	1500 ± 257 *
RMRe (LU)	1785 ± 184 *	1424 ± 165 *	1564 ± 247 *
RMRe (NA)	1906 ± 244 *	1442 ± 82 *	1622 ± 280 *
RMRe (CU)	1652 ± 134 *	1307 ± 102 *	1441 ± 204 *
RMRe (GO)	1735 ± 216 * (*n* = 31)	1400 ± 173 * (*n* = 40)	1546 ± 254 * (*n* = 71)

* Significant difference between measured and estimated RMR (*p* < 0.05). Note: Abbreviations: RMRm = resting metabolic rate measured; RMRe = RMR estimated by equation; HB = Harris and Benedict [8]; LU = Lührmann et al. [14]; NA = Nachmani et al. [10]; CU = Cunningham [9]; GO = Gougeon et al. [16].

**Table 4:** Percentage of subjects whose RMR was accurately estimated (within 10% of RMRm) underestimated (<10%RMRm) and overestimated (>10%RMRm).

Equation	Males (*n* = 35)			Females (*n* = 55)			Total (*n* = 90)		
	<10% RMRm (%)	>10% RMRm (%)	Accurate [%)	<10% RMRm (%)	>10% RMRm (%)	Accurate (%)	<10% RMRm (%)	>10% RMRm (%)	Accurate (%)
HB	42.9	0	57.1	65.4	0	34.6	56.7	0	43.3
LU	22.9	5.7	71.4	49.1	0	50.9	38.9	2.2	58.9
NA	5.7	22.9	71.4	41.8	7.3	50.9	27.8	13.3	58.9
CU	57.1	2.9	40.0	81.8	1.8	16.4	72.2	2.2	25.6
GO	41.9	0	58.1 (*n* = 31)	52.5	0	47.5 (*n* = 40)	47.9	0	52.1 (*n* = 71)

Note. Abbreviations: RMRm = resting metabolic rate measured; HB = Harris and Benedict [8]; LU = Lührmann et al. [14]; NA = Nachmani et al. [10]; CU = Cunningham [9]; GO = Gougeon et al. [16].

## Data Availability

The data presented in this study are available on request from the corresponding author. The data are not publicly available because the main results of the CEV-65 trial were not summarized and analyzed yet.
